# Why you may need a neurologist to see a comatose patient in the ICU

**DOI:** 10.1186/s13054-016-1372-8

**Published:** 2016-06-20

**Authors:** Eelco F. M. Wijdicks

**Affiliations:** Mayo Clinic, 200 First Street SW, Rochester, MN 55905 USA

**Keywords:** Comatose patient, Postcardiopulmonary resuscitation, Spontaneous eye movements, Traumatic brain injury, Prognostication

## Abstract

This commentary summarizes the value of a neurologist in the diagnosis and prognostication of coma. Evaluating coma is inherently complex, and neurologic consultation and management can be useful. We often find that management changes after a neurologic consultation.

## Background

Comatose patients in medical or surgical ICUs cause trepidation in the ICU team, but patients are not always evaluated by neurologists. Such consultation is probably contingent on the following five perceptions: an evolving situation that requires neurologic expertise; something might be missed; an unusual computed tomography (CT) scan that does not appear to explain the condition; movements that could indicate seizures with an ambiguous electroencephalogram (EEG) result; and the patient’s condition looks grim but needs corroboration, and the family may request a neurologic opinion. Neurologic consultations are most often requested when patients remain comatose after cardiopulmonary resuscitation (CPR), although in some institutions evaluation (and decisions) might be done entirely by the ICU team. Failure to awaken after surgery or after extended sedation has been discontinued is another typical example that triggers a request.

Evaluating coma is inherently complex, and the value of a neurologic consultation and management can be appreciated. There are also situations in which consults are highly productive; for example, cerebral fat embolization syndrome, an epidural spinal abscess in a comatose septic patient, and posterior reversible encephalopathy syndrome—all situations initially puzzling to the intensivist. We often find that management changes after a neurologic consultation [1]. Here are some reflections.

### Neurologic examination is more than a coma scale

**Fig. 1:**
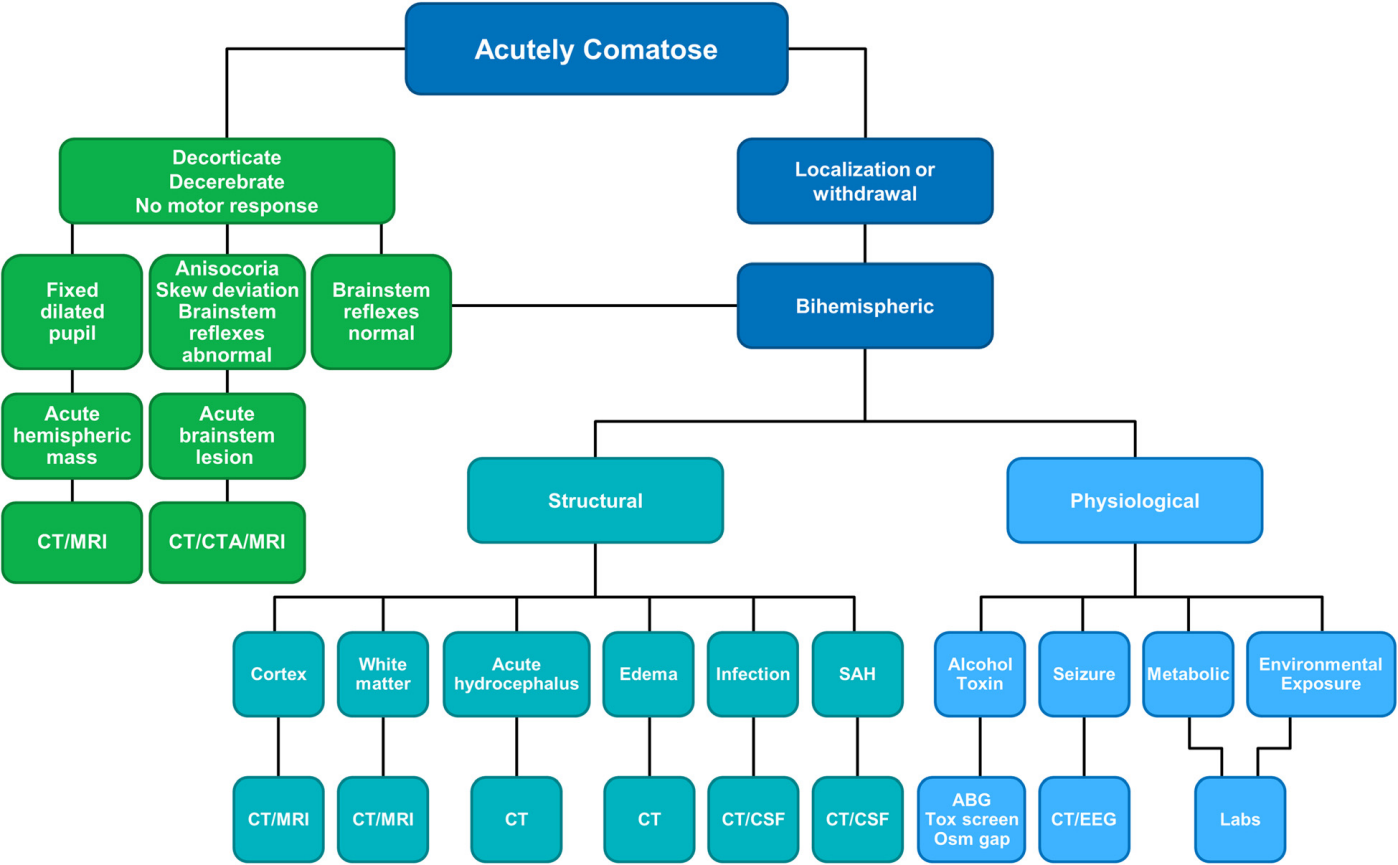
Initial thoughts on coma in the ICU. This algorithm is a simplification of clinical practice. Localization and withdrawal motor responses are most probably not associated with brainstem involvement, and therefore the dichotomy is made. Once abnormal brainstem reflexes are found, two options are likely—acute hemispheric mass or acute brainstem lesion. Bihemispheric injury is structural or physiological and further differentiated into specific locations and suggestions for tests. *ABG* arterial blood gas, *CSF* cerebrospinal fluid, *CT* computed tomography, *CTA* computed tomography angiography, *EEG* electroencephalogram, *SAH* Subarachnoid hemorrhage

The Glasgow Coma Scale (GCS) is a trauma tool and has made a major difference in communication to neurosurgeons [2]. The GCS is not a neurologic examination. Important elements in neurologic examinations, such as spontaneous eye movements, brainstem-reflex testing, and assessment of abnormal movements and tone, are not included, and these assessments are potentially relevant. The reliability of the GCS is questionable [2]. Alternatively, the FOUR Score provides considerably more useful information but is also not a full neurologic examination of the comatose patient [3]. Many subtleties may swing one way or another, but initially in a comatose patient, the motor responses and brainstem reflexes can be considered together and lead to decisions on tests that narrow the probable causes of coma If any, it is crucial to identify an embolus to the basilar artery which can be removed endovascularly (Fig. 1) [4].

### Confounders are found everywhere

Evaluating coma has not become any easier. The analgosedative protocols have improved care but have not been “friendly” to neurologists [5]. Drugs linger particularly in patients treated with targeted temperature management or in those with multiorgan failure [6]. Neurologists can determine whether certain signs are more suggestive of new structural injury than drug-induced dysfunction. It is no exaggeration to say that most misjudgments of coma occur when there has been prior drug use or sedation.

### Not all pupils are alike

Most literature compares clinical examination of pupillary reflex with quantitative pupillometry (often comparing nursing staff with a device [7]), but ICU studies fail to describe pupil size with pupil light reflex such as mid-position pupils or, the most important localizing finding, anisocoria [8]. We care less about very wide or pinpoint pupils. Extremely wide pupils often respond and are medication induced [9]. Pinpoint pupils often do not respond, artifactually, and are also commonly opioid related.

### Eye movements are good indicators of injury

Spontaneous eye movements are underdiagnosed and underappreciated. Many of them indicate severe structural brain injury, although they do not specifically localize, other than to diffuse cortical injury. Important spontaneous eye movements are ping-pong eye movements, vertical nystagmus, ocular bobbing, but also vertical conjugate eye deviation. In a prospective study on post-CPR coma, specifically targeted to examine eye movements, eye deviation downward or upward deviation appeared in nearly one in two patients. Upward deviation is often followed by downward deviation and a poor prognosticating sign [10, 11].

### Not all that seizes is a seizure

Many critically ill patients move repeatedly, and not all shivering is myoclonus. In fact, shivering, rigor, or nonsustained clonus is often misinterpreted as myoclonus. Myoclonus status epilepticus is an unusual presentation, often seen after prolonged CPR or exsanguination, and is vigorous and forceful, with jerks involving all four limbs and significant facial distortions, all in association with upward eye jerks.

### Could we practice with fewer EEGs"?

It is not common to diagnose status epilepticus in an acutely comatose patient without any evidence of a prior seizure, history of seizures or major predispostion for seizures. Or could we practice, at least, with fewer EEGs? The circumstances surrounding critical illness could make patients more vulnerable to seizures; however, while few patients in the ICU have seizures, many more undergo an EEG. Moreover the interpretation of abnormal EEG,s in these patients remains far from accurate. If the published studies are true-and I think they are-even if nonconvulsive status epilepticus is found....etc nonconvulsive status epilepticus is found, management of nonconvulsive status may not necessarily lead to better outcome if associated with irreversible cortical injury. EEG's may indicate prognosis but only with burst-suppression or flat background in an undrugged patient but generations of neurologists have known that.. But EEG in a comatose patient should never be used to make important decisions on care.

### Toxic metabolic encephalopathies are so yesterday

Elevated ammonia or blood urea nitrogen values seldom correlate with neurologic examination. Even if we think a correlation exists, nephrologists and hepatologists often deny it. For nearly half a century, patients who failed to awaken, were stuporous, or were not clearly delirious were labeled as having “toxic metabolic encephalopathy.” Some may be delirious. Some may have posterior reversible encephalopathy syndrome, cefepime toxicity, or simply metabolize drugs slowly. In many we simply do not know [12, 13].

### Neurologists know when to call a neurosurgeon or neurointerventionalist

Traumatic brain injury may require neurosurgical intervention, and this may not be clear on admission. Contusions and subdural hematomas may have delayed presentations [14]. Ischemic strokes do occur in cardiovascular surgical ICUs. With some of these patients, the onset time is tentatively known, and a CTA and CT perfusion can determine whether endovascular clot retrieval is warranted.

### Finally

We may bring closure when it comes to prognostication. We also—more frequently—say we do not know what the future holds. This part of consultative neurology is difficult and not for everyone. Assertively clarifying the reality to the patient’s family easily takes a toll both on neurologists and families witnessing their loved one’s neurologic state. We must avoid a poor outcome due to our self-fulfilling misjudgment or, worse, our coercion. We also must avoid analysis paralysis or creating false hope. Often, with decades of experiences, it is not so much probabilistic logic but more of “we know when we know.”

## Abbreviations

CPR, cardiopulmonary resuscitation; CT, computed tomography; CTA, computed tomography angiography; EEG, electroencephalogram; GCS, Glasgow Coma Scale
