# Erratum to: Why you may need a neurologist to see a comatose patient in the ICU

**DOI:** 10.1186/s13054-016-1395-1

**Published:** 2016-07-08

**Authors:** Eelco F. M. Wijdicks

**Affiliations:** Mayo Clinic, 200 First Street SW, Rochester, MN 55905 USA

Unfortunately, the original version of this article [1] contained several typographical and grammatical errors, none of which affected the scientific content of the article. These have now been corrected in the original article.
